# The expression of embryonic globin mRNA in a severely anemic mouse model induced by treatment with nitrogen-containing bisphosphonate

**DOI:** 10.1186/s12878-016-0041-0

**Published:** 2016-02-11

**Authors:** Hirotada Otsuka, Jiro Takito, Yasuo Endo, Hideki Yagi, Satoshi Soeta, Nobuaki Yanagisawa, Naoko Nonaka, Masanori Nakamura

**Affiliations:** Department of Oral Anatomy and Developmental Biology, School of Dentistry, Showa University, 1-5-8 Hatanodai, Shinagawa-ku, Tokyo, 142-8555 Japan; Division of Molecular Regulation, Graduate School of Dentistry, Tohoku University, 4-1 Seiryo-machi, Aoba-ku, Sendai, 980-8575 Japan; Faculty of Pharmacy, International University of Health and Welfare, 2600-1 Kitakanamaru, Otawara-shi, Tochigi 324-8501 Japan; Department of Veterinary Anatomy, Nippon Veterinary and Animal Science University, 1-7–1 Kyonan-cho, Musashino-shi, Tokyo 180-8602 Japan

**Keywords:** Embryonic hemoglobin, Nitrogen-containing bisphosphonate (NBP), Extramedullary erythropoiesis, KLF1, Nucleated erythrocyte

## Abstract

**Background:**

Mammalian erythropoiesis can be divided into two distinct types, primitive and definitive, in which new cells are derived from the yolk sac and hematopoietic stem cells, respectively. Primitive erythropoiesis occurs within a restricted period during embryogenesis. Primitive erythrocytes remain nucleated, and their hemoglobins are different from those in definitive erythrocytes. Embryonic type hemoglobin is expressed in adult animals under genetically abnormal condition, but its later expression has not been reported in genetically normal adult animals, even under anemic conditions. We previously reported that injecting animals with nitrogen-containing bisphosphonate (NBP) decreased erythropoiesis in bone marrow (BM). Here, we induced severe anemia in a mouse model by injecting NBP injection in combination with phenylhydrazine (PHZ), and then we analyzed erythropoiesis and the levels of different types of hemoglobin.

**Methods:**

Splenectomized mice were treated with NBP to inhibit erythropoiesis in BM, and with PHZ to induce hemolytic anemia. We analyzed hematopoietic sites and peripheral blood using morphological and molecular biological methods.

**Results:**

Combined treatment of splenectomized mice with NBP and PHZ induced critical anemia compared to treatment with PHZ alone, and numerous nucleated erythrocytes appeared in the peripheral blood. In the BM, immature CD71-positive erythroblasts were increased, and extramedullary erythropoiesis occurred in the liver. Furthermore, embryonic type globin mRNA was detected in both the BM and the liver. In peripheral blood, spots that did not correspond to control hemoglobin were observed in 2D electrophoresis. ChIP analyses showed that KLF1 and KLF2 bind to the promoter regions of β-like globin. Wine-colored capsuled structures were unexpectedly observed in the abdominal cavity, and active erythropoiesis was also observed in these structures.

**Conclusion:**

These results indicate that primitive erythropoiesis occurs in adult mice to rescue critical anemia because primitive erythropoiesis does not require macrophages as stroma whereas macrophages play a pivotal role in definitive erythropoiesis even outside the medulla. The cells expressing embryonic hemoglobin in this study were similar to primitive erythrocytes, indicating the possibility that yolk sac-derived primitive erythroid cells may persist into adulthood in mice.

**Electronic supplementary material:**

The online version of this article (doi:10.1186/s12878-016-0041-0) contains supplementary material, which is available to authorized users.

## Background

Mammalian hematopoiesis occurs in two distinct waves, commonly referred to as primitive and definitive, that originate in the yolk sac and in the fetal liver and bone marrow (BM), respectively [1]. Yolk sac-derived primitive erythroid cells remain nucleated and enucleated terminally in the circulation, whereas definitive erythroid cells produced in the fetal liver are released into circulation after complete maturation [2–5]. The components of the globin tetramer are encoded by the α- and β-globin gene loci. There are 3 functional α-globins (ζ-, α1- and α2) and 4 β-globins (Ey-, βh1-, β1- and β2-) in mice [2, 6, 7]. Primitive erythrocytes express the embryonic complement of globin chains, which initially consist of ζ- and βh1-globins, followed by α1- and α2-, and Ey-globins at the primitive proerythroblast stage [2, 6, 7]. Definitive erythroid cells complete their maturation and enucleation at erythropoietic sites, the fetal liver and BM, where the adult complement of globin chains consists of α1-, α2- β1- and β2- are expressed. All of the globin genes in the α- and β-globin clusters are expressed in primitive erythroid cells, whereas definitive erythroid cells express only the adult globin genes [8].

The expression of globin genes is jointly regulated by elements in the promoter regions and an upstream enhancer region called, the locus control region (LCR) [5]. The TATA, CAAT and CACCC regulatory elements are found within the promoters of all globin genes. A variety of nuclear factors that are involved in transcriptional regulation have been suggested to be related to globin gene expression and switches between them [9, 10]. Members of the KLF (Kruppel-like factor) family are known as an essential transcription factor that bind GC-rich sequences, such as the CACCC element, to regulate the biological dynamic during development [11]. KLF1 (EKLF: erythroid Kruppel-like factor) is a member of the KLF family that plays an essential role in erythropoiesis and is involved in the expression of β-like embryonic globin by binding to its promoter region [12, 13]. KLF2 has been found to regulate biological activity in various tissues and to enhance the expression of β-like embryonic globin [14, 15].

All hematopoietic cells are derived from hematopoietic stem cells (HSC) that develop into mature lineage cells during definitive hematopoiesis [2, 5]. Definitive erythropoiesis requires a specific microenvironment that contacts stromal cells; these groups of cells are called “erythroblastic islands”. Macrophages directly contact erythroid progenitors in the BM and fetal liver via several types of adhesion molecules, including EMP, α_V_ integrin and VCAM-1, which participate in their differentiation, survival and maturation during erythropoiesis [16–19]. These central macrophages may also be an important source of cytokines, particularly erythropoietin (EPO), which supports the maturation of erythroid cells [20, 21]. However, primitive erythropoiesis occurs without macrophages and some cytokines, such as SCF and EPO, are essential for definitive erythropoiesis [3]. These results indicate that erythroblast-macrophage interactions might be specifically essential for definitive erythropoiesis.

Bisphosphonates (BPs) are potent inhibitors of osteoclast-mediated bone resorption and are used as therapeutic agents against bone resorptive disorders such as osteoporosis and metastatic bone diseases [22, 23]. The use of nitrogen-containing bisphosphonates (NBPs) results in strong anti-bone resorptive effects that are much more potent than the effect of non-nitrogen-containing bisphosphonates (non-NBPs). In addition, NBPs have inflammatory side effects including fever, jaw osteomyelitis, osteonecrosis and extramedullary erythropoiesis [24–26]. Our previous study reported that a single injection of a relatively large dose of the NBP into mice inhibited bone resorption by osteoclasts, induced extramedullary erythropoiesis by depleting resident BM macrophages and increased the number of granulocytes in the peripheral blood [26]. Moreover, the injection of an NBP into splenectomized mice induced extramedullary erythropoiesis without anemia and alterations in EPO concentrations in the liver [27]. Phenylhydrazine (PHZ) is used to experimentally induce hemolytic anemia in laboratory animals [28]. The mechanism by which it causes anemia is RBC lipid peroxidation [29]. These results lead to suggest the change of erythropoiesis in the medullary and extramedullary hematopoietic sites by treatment with both NBP and PHZ, which indirectly inhibits erythropoiesis and directly disrupts erythrocytes.

In this study, we established a model of critical anemia in mice through the combined use of NBP with PHZ and analyzed the expression pattern of hemoglobin as well as changes in erythropoiesis and erythropoiesis-regulated factors in medullary and extramedullary erythropoietic sites.

## Methods

### Animals

**Fig. 1:**
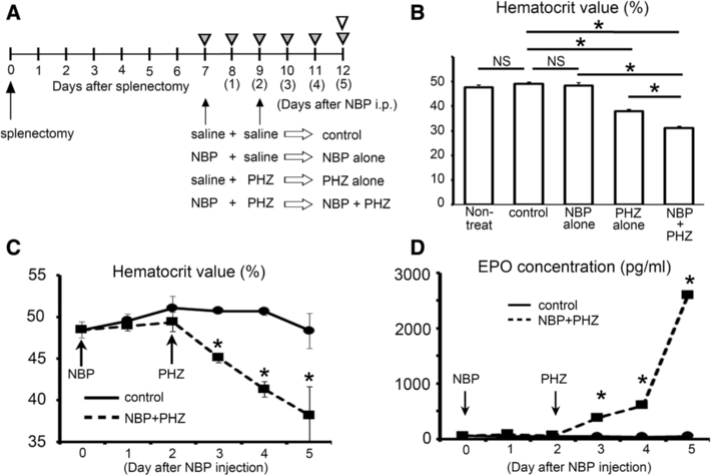
Experimental schedule and the analysis of peripheral blood. **a** The schema of the experimental schedule. Blood sample were collected every day (gray arrowhead), and tissue samples were obtained at 5 days after NBP injection (white arrowhead). Arrows indicate the time of the treatment (control, NBP alone, PHZ alone or NBP + PHZ). **b** The hematocrit values of all groups at 5 days after treatment. No change was observed by treatment with NBP alone, significant reduction were observed by PHZ treatment. Moreover, serious anemia was induced in the animals treated with both NBP and PHZ (NBP + PHZ). **c** The hematocrit values in the control animals and the animals treated with both NBP and PHZ (NBP + PHZ). Treatment with PHZ and NBP resulted in a significant reduction in hematocrit values after PHZ injection, which occurred 3 days after the NBP injection was detected. **d** The EPO concentration in the serum. The PHZ and NBP group (PHZ + NBP) had significantly increased EPO concentrations compared to the control. The error bars indicate the standard error of the mean (SEM)

Sixty female BALB/c mice (6-week-old) were obtained from Sankyo Laboratories (Tokyo, Japan), and were fed under specific pathogen-free conditions. The mice were anesthetized with an intraperitoneal injection of sodium pentobarbital (50 mg/kg) and performed splenectomies as described in previous reports [27]. The mice were divided into 5 group: a non-treatment group (5 mice), a splenectomized control group (20 mice), a splenectomized and NBP treatment group (10 mice), a splenectomized and PHZ treatment group (5 mice), and a splenectomized and both NBP and PHZ treatment group (20 mice). NBP (40 μmol/kg) or sterile saline was intraperitoneally injected into splenectomized mice 7 days after splenectomy. Two days after NBP injection, the mice were treated with PHZ (50 mg/kg) or saline, and they were killed 3 days after the PHZ or saline treatment (Fig. 1a). The NBP used in this study was 4-amino-1-hydroxybutylidene-1, 1-bisphosphonate (AHBuBP), which was prepared as previously described [24, 26, 27].

All experimental protocols used in this study were reviewed and approved by the Animal Care Committee of Showa University (Permit Number: 14043).

### Blood analyses

Peripheral blood samples were collected and centrifuged (10000 × *g*) to determine hematocrit values, and serum was then separated using centrifugation (1000 × *g*). To obtain serum EPO measurements, we used an EPO Mouse ELISA Kit (R & D systems, Minneapolis, MN, USA) according manufacturer’s protocol, and whole blood was prepared in blood smears and performed the May-Grünwald Giemsa staining technique.

### Antibodies and other materials

The monoclonal antibodies used in this study were as follows: purified anti-mouse TER-119 and anti-mouse CD71, FITC-conjugated anti-mouse TER-119 and PE-conjugated anti-mouse CD71 antibodies were purchased from BD Pharmingen (San Diego, CA, USA). Normal rabbit IgG, biotinylated goat anti-rat IgG antibodies and an avidin-biotin complex kit (ABC Elite standard kit) were purchased from Vector Laboratories (Burlingame, CA, USA). The polyclonal antibodies for ChIP, anti-KLF1 and KLF2 assays were obtained from Abcam (Cambridge, UK), and an alkaline-phosphatase (AP)-conjugated anti-digoxygenin (DIG) antibody was purchased from Roche (Basel, Switzerland). Anti-mouse TER119 antibody microbeads were purchased from Miltenyi Biotec (Bergisch Gladbach, DE).

### Tissue preparation

The tissue samples were fixed in 4 % paraformaldehyde prepared in phosphate-buffered saline (PBS). Tibias were demineralized using 10 % ethylenediaminetetraacetic acid (EDTA). Demineralized tibia and soft tissues were washed in 20 % sucrose-PBS, embedded in O.C.T. compound (Sakura Finetek Japan, Tokyo, Japan), and quickly frozen in a mixture of acetone and dry ice. Frozen sections (8 μm thick) were cut, placed on SILANE-coated glass slides, and air dried.

### Flow cytometry

The cells isolated from BM, the liver and peripheral blood were obtained as previously described [27, 30], washed using FACS solution (1 mM EDTA, 0.2 % BSA and 0.1 % NaN_3_ in PBS), and incubated with FITC-conjugated anti-TER-119 and PE-conjugated anti-CD71 antibodies or rat IgG (isotype control) in 1%BSA in PBS. After washing, the cells, they were resuspended in FACS solution and analyzed using a FACS Verse flow cytometer (BD Bioscience, Rockville, MD, USA). The data were collected for 10,000 events, stored in list mode, and subsequently analyzed using the BD FACS Suite software (BD Bioscience, Rockville, MD, USA).

### Histology and immunohistochemistry

Some sections were stained with hematoxylin-eosin (HE). The remaining sections were rinsed in PBS and fixed in 0.3 % H_2_O_2_-methanol for 30 min. After several PBS washes, the sections were incubated in 5 % normal goat serum in PBS. These sections were then incubated with an anti-TER119 or anti-CD71 antibody (1:50). After several rinses with PBS, the sections were incubated with biotinylated goat anti-rat antibodies, followed by a solution containing avidin-biotin-horseradish peroxidase complex. After the sections were washed, they incubated with a mixture of a DAB detection kit (KPL, Gaithersburg, MD, USA). Hematoxylin was used for counterstaining.

### *In situ* hybridization (ISH)

ISH was performed as previously described [31]. Antisense probes were designed to detect murine *hba-x* (ζ-globin, accession no. NM_010405), *hbb-bh1* (βh1-globin, accession no. NM_489729) and *hbb-y* (Ey-globin, accession no. NM_008219). All accession numbers were obtained from the Entrez nucleotide database. The designed probes were labeled using digoxygenin (RNA DIG labeling kit; Roche, Basel, Switzerland). The samples were fixed with 4 % paraformaldehyde and 0.5 % glutaraldehyde and prepared as frozen sections. The frozen sections were washed with PBS, digested with 1 μg/ml Proteinase K, and hybridized in separate buffer solution (50 % deionized formamide, 2 × SSC, 10 % dextran sulfate, and 0.01 % sheared yeast tRNA) containing each probe at 1 μg/ml at 50 °C. After hybridization, the sections were washed in SSC and unreacted probes were ablated using RNase A (Wako, Osaka, Japan). The probes were visualized using an AP-conjugated anti-DIG antibody with NBT/BCIP used as the substrate (Roche, Basel, Switzerland).

### RT-PCR, chromatin immune precipitation and quantitative RT-PCR

Mononuclear cells were isolated from BM and the liver as previously described [27], and TER119-positive cells were collected using the MidiMACS system immunomagnetic separation method, (Miltenyi Biotec, Bergisch Gladbach, DE). Total RNA was isolated using an RNeasy Mini kit (QIAGEN K.K., Hilden, DE) and reverse transcribed into cDNA using a PrimeScript RT reagent Kit (Takara, Shiga, Japan). A chromatin immune precipitation (ChIP) was performed according to the manufacturer’s instructions (Ez-ChIP; Millipore, Billerica, MA, US). PCR analysis was performed using Takara ExTaq® (Takara, Shiga, Japan). The primer sequences and annealing temperatures used in PCR are listed in Table 1.

**Table 1:** Used primer sequence

Name	Sequence	Annealing(°C) Product size
*Actin*	F: 5’-GCGTGACATTAAAGAGAAGCTG-3’	60
	R: 5’-CTCAGGAGGAGCAATGATCTTG-3	376 bp
*Hba*	F: 5’-CTCTCTGGGGAAGACAAAAGCAAC-3’	55
	R: 5’-GGTGGCTAGCCAAGGTCACCAGCA-3’	334 bp
*Hbb1*	F: 5’-CACAAACCCCAGAAACAGACA-3’	55
	R: 5’-CTGACAGATGCTCTCTTGGG-3’	529 bp
*Hba-x*	F: 5’- CTGTCTGCTGGTCACAATGG -3’	55
	R: 5’- GGGAGGAGAGGGATCATAGC -3’	165 bp
*Hbb-bh1*	F: 5’-TGGACAACCTCAAGGAGACC-3’	55
	R: 5’- TGCCAGTGTACTGGAATGGA -3’	231 bp
*Hbb-y*	F: 5’-CTTGGGTAATGTGCTGGTGA-3’	55
	R: 5’-GTGCAGAAAGGAGGCATAGC-3	183 bp
*Klf1*	F: 5’-CCTCCATCAGTACACTCACC-3’	55
	R: 5’-CCTCCGATT TCAGACTCACG-3’	150 bp
*Klf2*	F: 5’-CCAAGAGCTCGCACCTAAAG-3’	55
	R: 5’-GTGGCACTGAAAGGGTCTGT-3’	155 bp
*Bcl11a*	F: 5’-AACCCCAGCACTTAAGCAAA-3’	55
	R: 5’-ACAGGTGAGAAGGTCGTGGT-3’	122 bp
*Gata1*	F: 5’- ACCACTACAACACTCTGGCG -3’	60
	R: 5’- CAAGAACTGAGTGGGGCGAT -3’	452 bp
bh1-globin promoter	F: 5’-GGACAGGTCTTCAGCCTCTTGA-3’	56
	R: 5’-CAGATGCTTGTGATAGCTGCCT-3’	123 bp
Ey-globin promoter	F: 5’-TGCTTCTGACACTCCTGTGATCA-3’	56
	R: 5’-GGGTTTTTTCCTCAGCAGTAAAGT-3’	79 bp

Quantitative RT-PCR (qRT-PCR) was performed using an Applied Biosystems7500 Fast Real-time PCR system (Applied Biosystems Inc., Foster City, CA, USA) The following program was used: 50 cycles at 95 °C for 3 sec and 60 °C for 30 sec. The expression of different globin genes was measured using control ct values, and the ct values obtained for actin expression were used for normalization. The primers and probes used for qRT-PCR (TaqMan® gene expression assay, assay ID: *Hba*, Mm00845395_s1, *Hbb1*, Mm01611268_g1, *Hba-x*, Mm00439255_m1, *Hbb-bh1*, Mm00433932_g1 and *Hbb-y*, Mm00433936_g1) were obtained from Applied Biosystems (Foster City, CA, USA).

### 2D electrophoresis

Hemoglobin was isolated from blood samples, and 2D electrophoresis was performed as previously described [32]. The samples obtained from the hemolytic blood solution were treated with a 2D sample kit (ATTO, Tokyo, Japan). The carrier ampholyte isoelectric focusing method was combined with 10 % SDS-PAGE according to instructions in the ATTO Technical Manual (http://www.atto.co.jp/kotsu_series.html), and the gels were then stained using silver. The gel images were observed using chemiDoc (Bio-Rad, Hercules, CA, USA) and analyzed using NIH image software.

### Statistical analysis

For quantitative data analysis, a *t*-test was used to determine differences between paired samples. A *p-*value of <0.05 was considered statistically significant.

## Results

### Blood analyses

We collected peripheral blood to evaluate hematocrit value, to measure the serum EPO concentrations and to observe erythrocytes in blood smears. No changes were observed in hematocrit values and serum EPO levels in animals treated with splenectomy and NBP alone compared to the values in the non-treatment group (Fig. 1b). In splenectomized mice treated with PHZ, a significant reduction in hematocrit values was observed compared to controls and to animals treated with NBP alone (Fig. 1b). However, treating animals with both NBP and PHZ (NBP + PHZ) induced more serious anemia than administering PHZ alone (Fig. 1b). In an analysis of time-dependent changes following NBP injection, the hematocrit values in animals treated with both NBP and PHZ (NBP + PHZ) were significantly reduced compared to control animals at 1-3 days after PHZ-treatment (Fig. 1c). The concentration of EPO in serum samples taken from mice treated with NBP and PHZ was also significantly increased at 1-3days after PHZ-treatment (Fig. 1d).

**Fig. 2:**
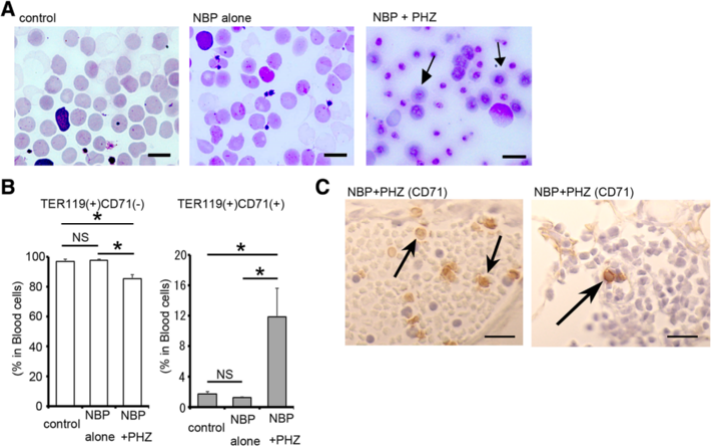
Morphological analysis of peripheral blood. **a** Blood smears stained with May-Grünwald Giemsa stain in control animals and the animals treated with NBP-alone (at 5 days after the NBP) and both NBP and PHZ (at 3 days after PHZ). Nucleated erythroid cells were easily detected in the NBP + PHZ mice (arrows). **b** Flow cytometry analysis of erythroid lineage cells in the peripheral blood at 5 days after NBP injection. The majority of cells in the control and NBP-alone groups were single-positive TER119 cells at 5 days after NBP injection, whereas the number of TER119 and CD71 double-positive cells was significantly increased by treatment with NBP and PHZ (NBP + PHZ) at 3 days after PHZ injection. **c** Immunohistochemical detection of CD71-positive cells in peripheral blood of splenectomized mice treated with NBP and PHZ (3 days after PHZ). CD71-positive reticulocytes were observed (arrows in left panel), and CD71-positive nucleated cells also existed in the blood vessel (arrows in right panel). Asterisks indicate statistical significance, and NS denote no significance versus the control. Bars indicate 10 μm (**a**) and 25 μm (**c**). The error bars indicate the SEM

The blood smears showed that only normal enucleated erythrocytes were observed in the control and NBP-only groups (Fig. 2a). In contrast, numerous nucleated erythrocytes and reticulocytes were observed in the peripheral blood of mice treated with both NBP and PHZ (Fig. 2a).

Flow cytometric analysis showed that the number of TER119 and CD71 double-positive cells was markedly increased in the mice treated with both NBP and PHZ, while almost erythrocytes were single-positive for TER119 in the other groups (Fig. 2b). In the blood vessel, some CD71-positive nucleated erythroid cells were observed in addition to CD71-positive reticulocytes (Fig. 2c).

These results indicate that splenectomized mice treated with both NBP and PHZ become critically anemic and display a significant increase in EPO levels, which enhances erythropoiesis. The results from the blood smears suggest the induction of abnormal erythropoiesis because nucleated erythrocytes are not normally observed in the blood in mammals except during the early embryonic stages.

### Detection of erythropoiesis in the BM

**Fig. 3:**
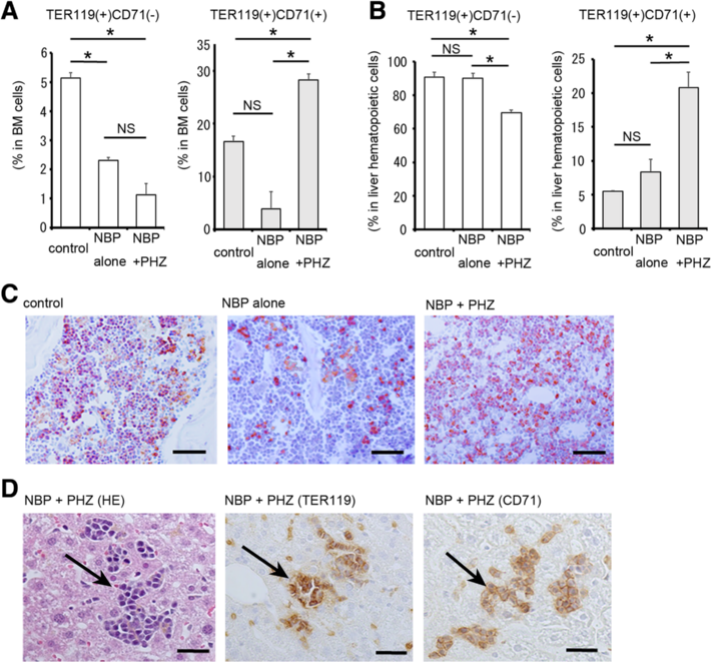
Erythropoiesis in the BM and liver. **a** Flow cytometric analysis of erythroid cells in the BM at 5 days after the treatment. In the controls animals, TER119 single-positive and TER119 and CD71 double-positive erythroid lineage cells comprised the majority of the cells. A significant decrease in the number of TER119-positive cells was induced by the NBP injection. In the animals treated with both NBP and PHZ (NBP + PHZ), the number of TER119 and CD71 double-positive (erythroid lineage) cells was markedly increased despite treated with NBP. **b** Flow cytometric analysis of erythroid cells in the liver at 5 days after the treatment. The number of TER119 single-positive cells was decreased in animals treated with both NBP and PHZ. TER119 and CD71 double-positive cells were rare in the control animals but were increased by treatment with NBP. The number of TER119/CD71-double-positive erythroblasts was decreased with the NBP treatment. The number of TER119/CD71 double-positive erythroblasts was higher in the group treated with both NBP and PHZ. **c** Immunohistochemical detection of TER119-positive cells in the BM at 5 days after the treatment. TER119-positive cells were significantly decreased by NBP treatment alone, but a decline in erythropoiesis was not detected in the group treated with both NBP and PHZ (NBP + PHZ). **d** Histological analysis of the livers of mice treated with NBP and PHZ at 5 days after NBP injection. The left panel shows HE staining. Mononuclear cells accumulated in the liver and formed numerous clusters (arrow). The middle and right panel show immunohistochemistry for TER119 and CD71, respectively. The cells that formed clusters in the liver were TER119- and CD71-positive erythroblasts (arrow). Bars indicated 50 μm (**c**) and 25 μm (**d**). The error bars indicate the SEM (**a** and **b**)

In the control BM samples, appropriately 20 % of all BM cells were TER119-positive, and three-fourth of the TER119-positive population of cells was TER119/CD71 double-positive (Fig. 3a). The TER119-positive erythroid lineage was significantly decreased by the injection of NBP (NBP alone) as a result of the elimination of resident BM macrophages, as previously described [26, 27]. However, the proportion of TER119-/CD71- double-positive cells in the NBP and PHZ group (NBP + PHZ) was significantly increased despite the use of NBP (Fig. 3a). The enhanced granulopoiesis and relative reduction of B-lymphopoiesis were observed in the BM of the animals treated with both NBP and PHZ (Additional file 1).

Many of the TER119-positive cells formed clusters in the control mice, as shown by immunohistochemistry (Fig. 3c). In the NBP-treated mice (NBP alone), fewer TER119-positive cells appeared to form into clusters in the BM (Fig. 3c). However, more TER119-positive cells were detected in the BM of the mice treated with both NBP and PHZ (Fig. 3c).

These results suggest that elevated levels of EPO supports the maturation of the erythroid lineage in the absence of BM macrophages and inhibits apoptosis during erythropoiesis in BM.

### Detection of erythropoiesis in the liver

In splenectomized mice, the liver is the main site of extramedullary erythropoiesis. Therefore, we next evaluated hepatic erythropoiesis using histological and immunohistochemical methods and flow cytometry. In control mice, TER119 single-positive cells were the most abundant, and CD71 double-positive cells were very rare (Fig. 3b). After the injection of NBP alone, the number of TER119 and CD71 double-positive cells was slightly increased (Fig. 3b). These results indicate that extramedullary erythropoiesis was induced in the liver. Furthermore, treatment with both NBP and PHZ induced a significant increase in TER119 and CD71 double-positive cells in the liver (Fig. 3b).

Histological analysis indicated the clusters of mononuclear cells in the livers of splenectomized mice treated with PHZ and NBP (Fig. 3d). These cells were TER119-positive and/or CD71-positive erythroblasts (Fig. 3d). Immunohistochmically, the clusters of granulocytes and lymphocytes could not be detected in the liver.

The injection of NBP and PHZ therefore induced extramedullary erythropoiesis in the liver and significantly increased the formation of erythroblast clusters compared to results in the control and NBP-only groups.

### Expression of embryonic globin mRNAs at hematopoietic sites

**Fig. 4:**
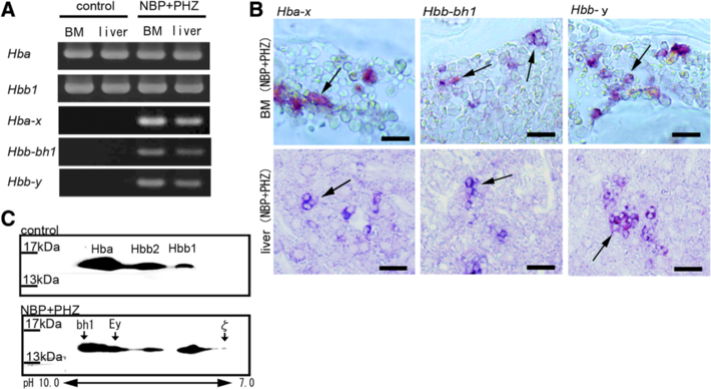
Expression of embryonic globins in the BM and liver. **a** RT-PCR analysis of globin mRNAs in the BM and liver. In control animals, only adult globins were expressed in the BM. Embryonic globins (*Hba-x*: ζ-, *Hbb-bha1*: βh1- and *Hbb-y*: Ey-globin) were expressed after treatment with both NBP and PHZ (NBP + PHZ) in the BM and liver. **b** *In situ* hybridization to detect embryonic globin. Some of hematopoietic cells in the BM and some cells that formed clusters in the liver expressed embryonic globins. These cells were diffuse in the BM. Bars indicate 20 μm. **c** 2D electrophoresis in the peripheral blood. The control showed three spots approximately 16 kDa in size. Three additional spots (arrows) appeared in the analysis of the blood of splenectomized mice that were treated with both NBP and PHZ (NBP + PHZ), with represented ζ-, βh1- and Ey-embryonic globin

To determine the expression pattern of hemoglobin subunits, we performed RT-PCR analysis on TER119-positive hematopoietic cells isolated from BM and the liver and collected using MACS (Miltenyi Biotec, Bergisch Gladbach, DE). The embryonic globins ζ-, βh1- and Ey- (*Hba-x*, *Hbb-bh1* and *Hbb-y*) and the adult globins α- and β major (*Hba* and *Hbb1*) were expressed in BM and the liver in the PHZ- and NBP-treated mice, whereas the TER119-positive cells in the control mice expressed only the adult globins (Fig. 4a). The expression level of embryonic globins in the liver was higher than the level in the BM, and *Hbb-y* was not detected in the control mice (Additional file 2). These results suggest that abnormal erythropoiesis may occur in this critical anemic model and that embryonic globins may be activated as a response to hypoxemia.

We next performed *in situ* hybridization to identify the cell clusters that expressed the embryonic globins ζ-, βh1- and Ey- (*Hba-x*, *Hbb-bh1* and *Hbb-y*) in the BM and liver of mice treated with both NBP and PHZ. We observed the expression of embryonic globins in the BM (Fig. 4b), but only a small number of cells expressed embryonic globin in the BM.

These results show that both embryonic-type and adult-type hemoglobin are co-expressed in clusters of cells in the BM and livers in splenectomized mice that were treated with NBP and PHZ and that definitive erythropoiesis could be of primary importance in the BM under these conditions.

### Hemoglobin fraction in the peripheral blood

We performed qRT-PCR to detect embryonic globins in the peripheral blood but did not detect the globins of embryonic-type in the control mice (Additional file 3). Therefore, we analyzed the pattern of hemoglobin expression in peripheral blood using 2D electrophoresis. While the molecular weights of embryonic and adult globins are very similar, the isoelectric points of embryonic globins differ from those of adult globins (Additional file 4). Control erythrocytes showed only 3 adult globin spots: α (α1 and α2 are the same), β major and β minor (Fig. 4c). The molecular weight of these globins were approximately 15.5-16.5 kDa, and the isoelectric points were different (Fig. 4c). However, erythrocytes from the NBP- and PHZ-treated mice showed 6 spots corresponding to 3 definitive globins and 3 embryonic globins (Fig. 4c).

These results indicate that embryonic globins were induced at both the mRNA and protein levels by treatment with both NBP and PHZ and that these globins can form embryonic or semi-embryonic type hemoglobin subunits.

### Expression of embryonic globin transcription factors

**Fig. 5:**
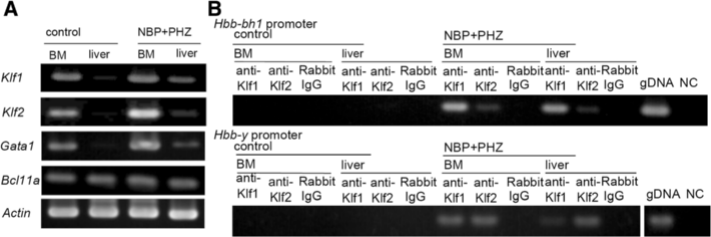
The expression of erythropoiesis-related transcription factors in the BM and liver. **a** The expression levels of erythropoiesis-related factors (*Gata1, Klf1* and *Klf2*) were up-regulated in the BM and liver after treatment with both NBP and PHZ (NBP + PHZ), but no change was observed in the expression of *Bcl11a*. **b** ChIP-PCR analysis of KLF1 and KLF2 promoter-binding region. KLF1 and KLF2 promoter-binding regions were detected using PCR amplification in splenectomized mice that were treated with NBP and PHZ but not in control animals. Genomic DNA (gDNA) was used as a positive control. NC indicates results of PCR performed without primers. Rabbit IgG was used as a negative control for ChIP

The expression of several embryonic globin transcription factors was analyzed using RT-PCR. The expression levels of *Klf1, Klf2* and *Gata1* were up-regulated, but the level of *Bcl11a* was unchanged in control animals and animals treated with NBP and PHZ (NBP + PHZ) (Fig. 5a). Because the expression of *Klf1* and *Klf2* was markedly increased, we used ChIP and PCR (ChIP-PCR) targeting of the embryonic globin promoters with anti-KLF1 or anti-KLF2 antibodies. DNA fragments that bound to KLF1 or KLF2 were used for globin promoter PCR. The results showed that binding of the βh1- and Ey-globin promoters bound KLF1 and KLF2 in the BM and livers of mice treated with NBP and PHZ (Fig. 5b). These results indicate that KLF1 and/or KLF2 regulate the transcription of embryonic β-like globins in the BM and liver. The expression patterns of *Bcl11a* suggests that the globin switching mechanism observed in this study might be different from the mechanism used during embryogenesis because Bcl11a plays an essential role in globin switching in normal ontogenic development of eythropoiesis.

### Extramedullary erythropoiesis in newly identified structures

Extramedullary erythropoiesis occurs in various organs, including the lungs, heart, thymus, and hemal nodes, under abnormal conditions.

**Fig. 6:**
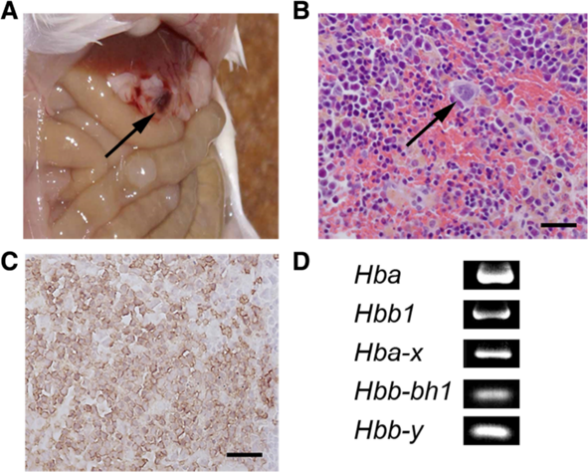
Extramedullary erythropoiesis in newly identified structures. **a** Macroscopic observation of the abdominal cavity. Wine-colored structures were observed in the omentum in splenectomized mice that were treated with both NBP and PHZ (arrow). **b** The histological analysis stained with H-E. Numerous hematopoietic cells including megakaryocytes (arrow), were observed in these structures. **c** Immunohistochemical detection of TER119-positive cells. Most of the cells in this structure were TER119-positive erythroblasts. **d** RT-PCR analysis to determine globin mRNA levels in these newly identified structures. Both adult and embryonic globin mRNAs were expressed. Bars show 25 μm (**b, c**)

In this study, we unexpectedly observed the formations of wine-colored miliary structures in the abdominal cavities of several mice that were treated with both NBP and PHZ (Fig. 6a). In histological examination, these structures were capsule-shaped and filled with many hematopoietic cells, including megakaryocytes (Fig. 6b). Immunohistochemical analysis showed that many of the mononucleated cells were TER119-positive erythroblasts (Fig. 6c). Moreover, the embryonic globin mRNA was detected in these structure (Fig. 6d). These results indicated that these structures were the site of induction for extramedullary erythropoiesis in this critically anemic mouse model.

## Discussion

Primitive and definitive erythroid cells display many differences that are consistent with the notion that they constitute distinct erythroid lineages. The two sequences of erythropoietic events that lead to the development of these cells never occur simultaneously in adults; therefore, embryonic globin is not expressed in adult mammals under normal conditions [2, 5, 9, 31]. However, in this study, we detected the expression of embryonic globins in genetically normal adult splenectomized mice under specific conditions including the the elimination of BM resident macrophages and the induction of critical anemia. Moreover, numerous nucleated erythrocytes appeared in the peripheral blood, and some erythroid cells were found to be CD71-positive. These cells were very similar to primitive erythrocytes [32]. Although certain diseases such as thalassemia and some knockout models that contain deletions in some important hemoglobin switching genes are associated with the expression of embryonic globins after birth, there are no reports showing that wild type adult mice express embryonic globins [32–34]. Therefore, the present study contributes a novel experimental model that can be used to analyze the expression of embryonic globins and the results of this study suggests that yolk sac-derived primitive erythroid precursors are maintained in adult mammals in this model.

Various transcription factors related to erythropoiesis and the regulation of hemoglobin expression have been identified [5, 11]. In this study, the up-regulation of *Klf1* and *Klf2* was detected in the BM and liver. Moreover, we also confirmed KLF1 and KLF binding to the promoter region of β-like embryonic globin. These results indicate that KLF1 and KLF2 regulate the expression of β-like embryonic globin. We detected the expression of *Gata1* and *Bcl11a* mRNAs in the BM and liver in splenectomized mice that were treated with both NBP and PHZ. A recent study has shown that *Bcl11a* expression is regulated by KLF1, suggesting an intricate mechanism in which the developmental regulation of β-like globin genes is coordinated by KLF1 and Bcl11a [35]. In fact, the conditional knock out of *Bcl11a* in mice resulted in a decrease in the expression levels of embryonic globins in the BM compared to levels in the E18.5 fetal liver [36]. In this study, *Bcl11a* was constitutively expressed in the BM and liver under normal conditions, and its expression levels were unchanged even in animals with anemia. These results suggest that Bcl11a may not be associated with the expression of embryonic globin in this study because the cells expressing embryonic globin contained decreased levels of Bcl11a and the cells that expressed adult globin showed no change in the expression of Bcl11a. In addition, Bcl11a is essential for normal lymphocyte development mice.

During embryogenesis, the embryo is exposed to hypoxemia. Therefore, primitive hemoglobin has a higher affinity for oxygen than adult hemoglobin because it must provide all oxygenation to the body, and changes in the partial pressure of oxygen has been associated with globin switching [37, 38]. Our data suggest that the expression of embryonic globins might be more effectively rescuing the hypoxemia that is associated with critical anemia than the enhanced production of normal definitive erythroid cells. The hypoxic response is controlled by two transcriptional regulatory complexes called hypoxia inducible factors (HIFs). These link iron homeostasis and erythropoiesis by targeting EPO [5, 39]. Although a direct association between the HIFs and the expression of embryonic globins has not been described, EPO has been suggested to support primitive hematopoiesis and to increase the expression of embryonic globin during embryogenesis [40, 41]. Therefore, the high EPO concentration caused by hypoxia might be partially responsible for the expression of embryonic globins in this study.

We confirmed that there were differences in expression patterns between the BM and liver, suggesting that cells in the liver originate in a different microenvironment, via a different mechanism and/or from a cell origin that is different from that in the BM. In fact, some hematopoiesis-related factors show different expression patterns between the E18.5 fetal BM and liver [42]. Our ISH data indicate that a relatively large number of cells expressed embryonic globins in the liver whereas only a few cells expressed these mRNAs in the BM. qRT-PCR analysis also showed that the expression level of embryonic globin in the liver was higher than level in the BM (Additional file 2). Erythropoiesis in the BM may primarily produce definitive erythroid cells, and the primitive-type may originate from a small population of hematopoietic cells. The liver is associated with fetal hematopoiesis, including primitive erythropoiesis [43]. These results may be related to the availability for quick induction of extramedullary hematopoiesis and the formation of a niche for primitive erythropoiesis in the liver.

Resident macrophages act as pivotal stromal cells during definitive erythropoiesis. In fact, erythropoiesis was inhibited by the elimination of BM resident macrophages in our previous studies [26, 27]. In addition, macrophages perform essential roles even in extramedullary erythropoiesis and abnormal erythropoiesis, such as that observed in β-thalassemia, whereas primitive erythroid cells do not require macrophages in the stroma [44]. An ISH study indicated that some TER119-positive colonies were composed of both embryonic globin-expressing and -non-expressing cells. In the early stage of the transition from primitive to definitive hematopoiesis, primitive hematopoietic cells migrate to the fetal liver, contact macrophages, and continue to express embryonic type globins within a short period of time. In our experiments, some of the TER119-positive cells did not express embryonic globin mRNAs. These results did not clearly indicate the origin of the hematopoietic cells expressing embryonic globins, but they do suggest that the expression of embryonic globins in this study could have been caused by both the depletion of macrophages and stimulation with an acute hypoxemia.

In this study, we were unable to define the origin of the erythroid cells that expressed embryonic globins. Because some tissue macrophages originate in the yolk sac and then proliferate in peripheral tissues [45, 46], we were unable to eliminate the possibility that these cells were derived from primitive hematopoietic cells and that these primitive erythroid precursor cells might be reactivated by the elimination of stroma cells and the presence of hypoxic stress. Further studies will be necessary to determine the origin of these cells.

New structures were unexpectedly induced in the peritoneum and/or omentum of some of the splenectomized mice that were treated with both NBP and PHZ (Fig. 6). These were wine-colored structures that resembled hemal nodes and showed active hematopoiesis. Extramedullary erythropoiesis is induced in various organs by certain conditions such as acute anemia, NBP injection, myelofibrosis, and blood cancer [26–28]. The induction of hemal node-like structures could be caused by insufficient hematopoiesis in the BM and the liver. Further study is required to clarify the mechanism that cause the induction of these newly hematopoietic organs.

The results showing that a relatively large number of erythroblasts form clusters and nucleated erythrocytes in the peripheral blood suggest the existence of primitive erythroid cells in the mice. These cells were large, nucleated erythrocytes. The presence of embryonic or fetal hemoglobin has been suggested in hematopoietic progenitors in human peripheral blood, but this does not explain the expression of these hemoglobin in hematopoietic sites [47, 48]. Several reports have also demonstrated the expression of embryonic or fetal globins *in vitro* [49, 50]; however, our study has shown for the first time, the simultaneous expression of embryonic and adult globins in erythrocytes in genetically normal mice. These results contribute to our understanding of normal and disease-related hematopoiesis.

## Conclusions

We established a severely anemic mice model by the sequential treatment with NBP and PHZ. This model showed the emergence of nucleated erythrocytes in peripheral blood and the expressions of embryonic types of hemoglobin in hematopoietic sites. Our results might indicate the flexible property of hematopoiesis and the possibility that yolk sac-derived primitive erythroid cells may persist into adulthood in mice.

## Availability of data and materials

We please to share all data for all researchers.

## Additional files

Additional file 1:
**Proportion of hematopoietic cells in the BM and liver.** (DOC 49 kb) Additional file 2:
**Quantitative PCR for globin mRNA expression in the BM and liver.** (DOC 88 kb) Additional file 3:
**qRT-PCR analysis of globin mRNA expression in the peripheral blood.** (DOC 61 kb) Additional file 4:
**The molecular weights and isoelectric pointsof globin proteins.** (DOC 46 kb)

## Abbreviations

NBP: nitrogen-containing bisphosphonate
PHZ: phenylhydrazine
BM: bone marrow
EPO: erythropoietin
AP: alkaline-phosphatase
PBS: phosphate-buffered saline
EDTA: ethylenediaminetetraacetic acid
HE: hematoxylin-eosin
ISH: in situ hybridization
qRT-PCR: quantitative RT-PCR
ChIP: chromatin immunoprecipitation
