# Supplementary material for: The expression of embryonic globin mRNA in a severely anemic mouse model induced by treatment with nitrogen-containing bisphosphonate
Source: BMC Hematol. 2016 Feb 11;16:4. doi: 10.1186/s12878-016-0041-0 (PMC4751657; doi:10.1186/s12878-016-0041-0)
Supplement: Supplementary file 1 [file 12878_2016_41_MOESM1_ESM.doc]

	Gr1(+) (%±SE)	B220(+) (%±SE)	CD3(+) (%±SE)
**BM control**	56.8 ± 6.6	23.4 ± 0.9	0.75 ± 0.25
**BM NBP+PHZ**	74.35 ± 1.35	3.0 ± 0.4	0.4 ±0.3
**Liver control**	11.6 ± 3.0	40.97 ± 9.2	6.0 ± 1.4
**Liver NBP+PHZ**	20.55 ± 0.45	35.95 ± 1.95	5.45 ± 1.05

Additional File 1. Proportion of hematopoietic cells in the BM and liver.

The enhanced granulopoiesis and relative reduction of B-lymphopoiesis were observed in the BM in the animals treated with both NBP and PHZ.
