# Supplementary material for: The expression of embryonic globin mRNA in a severely anemic mouse model induced by treatment with nitrogen-containing bisphosphonate
Source: BMC Hematol. 2016 Feb 11;16:4. doi: 10.1186/s12878-016-0041-0 (PMC4751657; doi:10.1186/s12878-016-0041-0)
Supplement: Supplementary file 2 [file 12878_2016_41_MOESM2_ESM.doc]

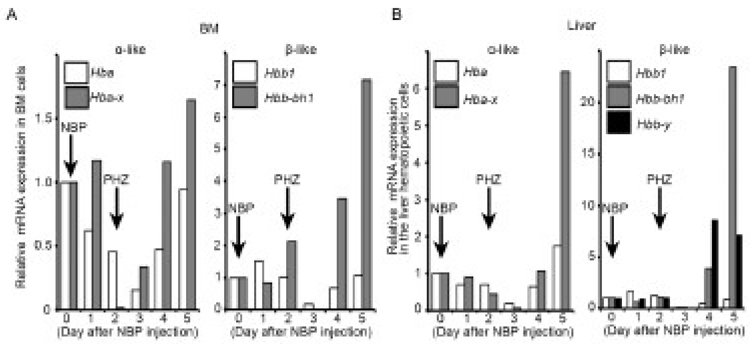

Additional File 2. Quantitative PCR for globin mRNA expression in the BM and liver.

Quantitative PCR to determine globin mRNA expression in the BM (A) and liver (B). The expression of different globin genes was measured using day 0 as the control ct value; ct values were obtained for actin expression and used for normalization.
