# Supplementary material for: The expression of embryonic globin mRNA in a severely anemic mouse model induced by treatment with nitrogen-containing bisphosphonate
Source: BMC Hematol. 2016 Feb 11;16:4. doi: 10.1186/s12878-016-0041-0 (PMC4751657; doi:10.1186/s12878-016-0041-0)
Supplement: Supplementary file 3 [file 12878_2016_41_MOESM3_ESM.doc]

	Hba	*Hbb1*	*Hba-x*	*Hbb-bh1*	*Hbb-y*
**Control**	-1.516	6.949	Undetected	Undetected	Undetected
**NBP+PHZ**	-3.913 (5.268)	-3.914 (1862.083)	14.067 (-)	12.43 (-)	13.58 (-)

n=3

Additional File 3. qRT-PCR analysis of globin mRNA expression in the peripheral blood

Real-time PCR analysis of globin mRNA expression in the blood of control animals and animals treated with NBP and PHZ (at 3 days after PHZ). The number denotes the ⊿CT mean values, where ⊿CT = Σ{(each globin CT)-(each actin CT)}/3. The number in parenthesis indicates the relative expression value against the control. Embryonic globin mRNAs were not detected in the control animals. NBP+PHZ denotes splenectomized mice that were treated with both NBP and PHZ.
