# Supplementary material for: The expression of embryonic globin mRNA in a severely anemic mouse model induced by treatment with nitrogen-containing bisphosphonate
Source: BMC Hematol. 2016 Feb 11;16:4. doi: 10.1186/s12878-016-0041-0 (PMC4751657; doi:10.1186/s12878-016-0041-0)
Supplement: Supplementary file 4 [file 12878_2016_41_MOESM4_ESM.doc]

	Name	MW (kDa)	Isoelectric point (pH)
**Primitive**	**ζ-globin**	16.235	7.06
	**βh1-globin**	16.484	8.69
	**Ey-globin**	16.127	7.90
**Definitive**	**α-globin**	15.085	7.97
	**β major globin**	15.840	7.13
	**β minor globin**	15.878	7.85

Additional File 4. The molecular weights and isoelectric points of globin proteins.
